# Numerical Simulation of the Temperature Field and Stress Evolution Behavior of a Hot-Rolled Seamless Steel Tube Under Temperature-Controlled Quenching

**DOI:** 10.3390/ma19122519

**Published:** 2026-06-11

**Authors:** Rui Zhang, Zhenlei Li, Dong Chen, Lijun Wang, Haijun Zhang

**Affiliations:** 1 School of Low-Carbon Energy and Power Engineering, China University of Mining and Technology, Xuzhou 221116, China; zhang.rui@cumt.edu.cn; 2 State Key Laboratory of Digital Steel, Northeastern University, Shenyang 110819, China; neu_chendong@163.com; 3 National Engineering Research Center of Coal Preparation and Purification, China University of Mining and Technology, Xuzhou 221116, China; zhjcumt@163.com; 4 School of Chemical Engineering, China University of Mining and Technology, Xuzhou 221116, China

**Keywords:** cooling intensity, temperature-controlled quenching, temperature distribution, compressive stress, tensile stress

## Abstract

The production process of hot-rolled seamless steel tubes often needs secondary heating for tempering treatment, resulting in high energy consumption and low production efficiency. Controlled cooling technology has been introduced into the production process. By controlling the self-tempering temperature, the residual temperature of the steel tubes is used to realize self-tempering, so as to achieve the purposes of energy savings, emissions reduction, cost reduction, and efficiency increase. This study investigated the evolution of temperature and stress fields in a seamless steel tube during temperature-controlled quenching. The size of the steel tube is 140 mm × 20 mm × 200 mm. The outer-wall cooling intensity and the self-tempering temperature were selected as the main variables, and the other process parameters remained constant. The temperature distribution and stress variation curves under different cooling intensities were obtained. The results showed that the greater the cooling intensity of the outer wall, the higher the temperature recovery of the outer wall. Under the fixed cooling intensity, the lower the self-tempering temperature, the lower the return temperature. In the thickness direction of the steel tube, there is a stress distribution of “internal tension and external compression”. Moreover, the greater the cooling intensity, the lower the self-tempering temperature and the greater the residual stress.

## 1. Introduction

Hot-rolled seamless steel tubes are important engineering products widely used in oil exploration, natural gas transportation, urban infrastructure, and other industrial applications. The manufacturing process involves piercing and rolling a tube billet into the required dimensions, followed by heat treatment to achieve the desired mechanical properties. Tempering is an indispensable process in off-line heat treatment. Tempering refers to the quenching workpiece being heated to the appropriate temperature again, held several times, and then slowly or quickly cooled. Generally, tempering is used to eliminate the internal stress in hardened steel parts and to avoid cracking of the workpiece [1,2,3]. The desired mechanical properties can be obtained by combining quenching and tempering. Steel tubes are usually cooled to room temperature during quenching, after reheating for the tempering treatment. This production process has a large energy consumption and low production efficiency. It is urgent to improve the production process and ensure product quality while reducing energy consumption.

**Figure 1:**
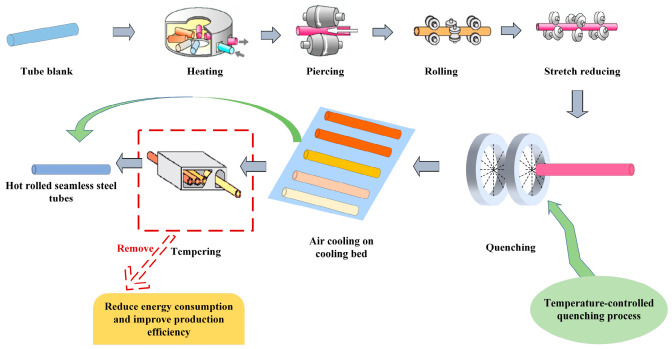
Production process of hot-rolled seamless steel tubes.

Controlled cooling technology is used to control the cooling rate to improve the microstructure and properties of steel [4,5,6]. The technology can also control the material quenching path, effectively using the waste heat of the quenched product to achieve self-tempering, and organically combine the quenching and heat treatment process. During the quenching process, the surface of the product reaches a lower temperature in a short time. However, there is still a higher temperature in the center, and the residual temperature is transferred to the quenched surface through heat conduction to achieve self-tempering. Compared with the traditional quenching and tempering process, self-tempering after quenching eliminates the need for reheating and tempering, further reducing the production process in Figure 1. It can achieve cost reduction and efficiency improvement, energy savings, and emissions reduction.

At present, temperature-controlled quenching technology has been successfully applied in the industrial production of strip and achieves a good application effect. The controlled cooling is affected by the material size, the quenching temperature, the self-tempering temperature, and the cooling intensity. The most important factors are the cooling intensity and the self-tempering temperature. Many scholars have conducted many investigations and research studies on it. Singh et al. [7] studied the effect of the nozzle shape on the cooling intensity by numerical simulation. They pointed out that the rectangular nozzle has the maximum mass flow and the best cooling effect at a fixed Reynolds number. Under the same mass flow rate, the cooling effect of the circular nozzle is better. N. NITIN [8] studied the heat transfer characteristics of a slit air jet impinging on a cylinder by computational fluid dynamics. It was found that the average Nusselt number increases with the increase in the Reynolds number, and the curvature of the cylinder surface and has little correlation with the jet distance. Pachput et al. [9] found that the number of circular jets required for uniform cooling increases with the increase in the cylindrical curvature D/d. In addition, the correlation is given between the Nusselt number at the stagnation point and the average Nusselt number. Moreover, the influence of tilt angle [10,11,12], cooling medium, and jet layout [13,14,15] on cooling intensity has also been widely reported.

Quenching is a thermal elastic–plastic problem inside a nonlinear material. The heat treatment process of materials is a complex process with multiple coupling fields of material constitution, temperature field, microstructure field, and stress field. The thermal stress of the workpiece is caused by the temperature difference between one part of the workpiece and another during quenching. By controlling the self-tempering temperature, the quenching stress can be reduced. The quality of products can be improved by self-tempering. Wang et al. [16] pointed out that reducing the quenching time can reduce the quenching stress and avoid the workpiece cracking. Chen et al. reported that forging heat quenching is used for the forgings of automobile front axles. The self-tempering temperature is controlled to be above 150 °C. Then, high-temperature tempering is performed. It can improve the strength of the forgings and the plastic toughness. Pan et al. [17] proposed the water–air alternating time-controlled quenching (ATQ) process, which can significantly reduce the surface cracking tendency and improve the mechanical properties of large-section forgings after water quenching for a certain period of time. Peng Qing [18] analyzed the effect of different self-tempering temperatures on the microstructure and mechanical properties of steel bars. The results showed that the flexural strength ratio decreased with increasing self-tempering temperature. By comparison, they found that a self-tempering temperature between 500 °C and 560 °C can make the best-performing steel bars. Li et al. [19] investigated an impingement–effusion cooling system using fluid–thermal–structural coupled simulations and reported that increasing the blowing ratio improved the overall cooling efficiency, while aggravating the thermal and thermo-mechanical stress concentrations around the film holes. He et al. [20] numerically analyzed the effect of local cooling on welded joints with non-uniform thicknesses and demonstrated that local cooling reduced tensile stresses in the weld zone, homogenized the stress distribution within the heat-affected zone (HAZ), and decreased the angular distortion by 18.0% and 26.7% in the thin and thick plates, respectively. Xi et al. [21] employed a coupled aerodynamic–thermal and thermo-mechanical numerical approach to evaluate the cooling performance of gas turbine stator blades. Their results showed that increasing the turbulence intensity of the cooling fluid from 0.05 to 0.15 improved the cooling effectiveness by 2.06% and reduced the maximum thermal stress by 1.12%. Qi et al. [22] investigated the evolution of residual stresses during the laminar cooling process of hot-rolled dual-phase steel and found that reducing the initial temperature difference, adopting medium-wave or quarter-wave plate profiles, and applying sparse or ultra-fast cooling strategies could effectively alleviate excessive compressive stresses at the plate edges. However, non-uniform stress distributions along the plate width were observed during the later stages of cooling. For multiphase steel tubes, Leitner et al. [23] proposed a controllable cooling design strategy for tailoring residual stress and phase distributions. Their study demonstrated that the residual stress distribution was independent of the tube diameter. While external intensive cooling alone generated a residual stress field with fixed characteristics, combined internal–external cooling enabled flexible and precise control of both residual stresses and phase distributions by adjusting the cooling parameters such as the water flow rate and the cooling duration. These studies indicate that cooling conditions play a critical role in determining the thermal and stress responses of metallic components. Nevertheless, most existing investigations have focused on plates, welded structures, and turbine blades, whereas the coupled evolution of temperature and stress fields during the cooling of hot-rolled seamless steel tubes, particularly under different internal cooling configurations, remains insufficiently understood.

In our previous study [24], the influence of cooling intensity on quenching stress evolution in hot-rolled seamless steel tubes was investigated. However, the temperature-controlled cooling process was not considered, and the reheating phenomenon associated with self-tempering was not analyzed. In addition, the coupling relationship between cooling intensity, self-tempering temperature, temperature evolution, and stress development was not systematically clarified. Therefore, the present study extends the previous work by introducing self-tempering temperature as an additional process parameter and investigating both temperature evolution and stress development during temperature-controlled cooling. Particular attention is paid to the temperature recovery phenomenon, the stress redistribution behavior, and the influence of the cooling intensity and the self-tempering temperature on the final stress state. The results provide further guidance for optimizing temperature-controlled cooling processes in hot-rolled seamless steel tube production.

## 2. Methods

### 2.1. Material and Physical Model

**Figure 2:**
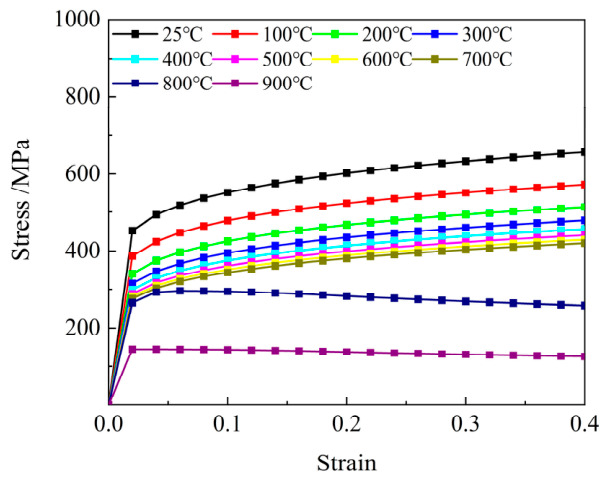
Stress–strain curve of 310S stainless steel.

**Figure 3:**
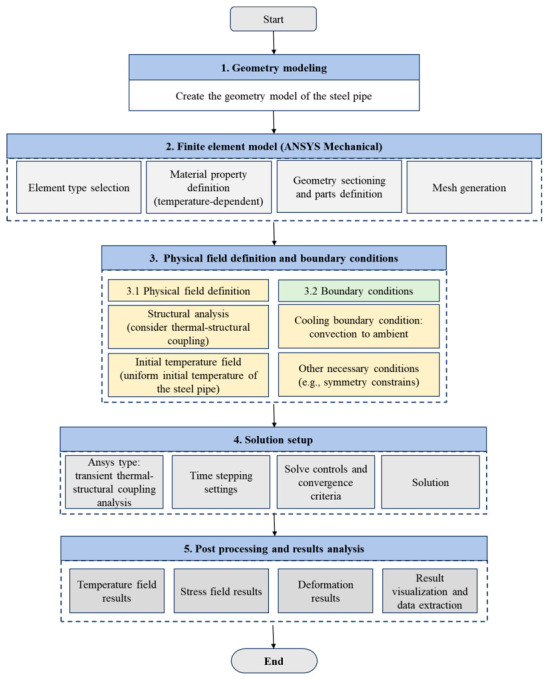
Schematic diagram of the calculation process.

In this study, ANSYS 2022 is used to simulate the temperature field and stress field of the hot-rolled seamless steel tube during temperature-controlled quenching. The size of the steel tube is 140 mm × 20 mm × 200 mm. Since the steel tube is an axisymmetric model, a 1/8 model is used to reduce calculation time. AISI 310S stainless steel was selected as the model material, and its chemical composition is listed in Table 1. It remains fully austenitic during cooling and does not undergo phase transformation. This characteristic allows the influence of temperature gradients on thermal stress evolution to be investigated independently without interference from transformation-induced strains. The purpose of this study is not to reproduce the complete metallurgical behavior of commercial low-alloy seamless steel tubes but rather to establish a fundamental thermo-mechanical model for analyzing temperature-controlled cooling and stress evolution. The thermophysical parameters and stress–strain curves of 310S stainless steel are shown in Table 2 and Figure 2. The thermal properties of 310S stainless steel are input by linear interpolation. The calculation process is shown in Figure 3.

To simplify the calculation process, the following assumptions are made:

- The initial temperature of the steel tube is evenly distributed and kept constant.
- There is no residual stress in the initial state of the steel tube.
- The seamless steel tube is evenly cooled during the quenching process.

### 2.2. Calculation Equation

The three-dimensional thermal differential equation in a cylindrical coordinate system is used to calculate the temperature field. To determine the unique thermal differential equation, the initial and boundary conditions, namely the definite solution conditions, are required. The initial condition refers to the temperature distribution of the object area at the initial time (T_0_). In addition, the third type of boundary condition was used to set the temperature of the cooling medium and the surface heat transfer coefficient during the quenching process of the steel tube [25,26,27].

(1) $$\frac{\partial }{\partial r}\left(\lambda r\frac{\partial T}{\partial r}\right)+\frac{1}{r^{2}}\frac{\partial }{\partial \varphi }\left(\lambda \frac{\partial T}{\partial \varphi }\right)+\frac{\partial }{\partial z}\left(\lambda \frac{\partial T}{\partial z}\right)+\dot{\Phi }=\rho c\frac{\partial T}{\partial t}$$

(2) $$T{|}_{t=0}=T_{0}$$

(3) $$\lambda \left(\frac{\partial T}{\partial n}\right)=h\left(T_{w}-T_{f}\right)$$

where *λ* is the thermal conductivity coefficient in W/(m·°C); *ρ* is the material density in W/(m·°C); *c* is specific heat capacity of material; *T* is the temperature of the steel tube in °C; t is time in s; and $\dot{\Phi }$ is the internal heat source derived from the heat release of phase transition during quenching. No phase transition occurs in the simulation; herein, the value is 0. *r* is the radial distance of the steel tube; *φ* is azimuth; Z is the height in mm; n is the normal of the steel tube heat exchange surface; *T_w_* and *T_f_* are the wall temperature and the fluid temperature in °C, respectively; and *h* is the surface heat transfer coefficient in W/(m^2^·°C).

In this finite element simulation, the initial temperature of the steel tube is 900 °C, and the temperature of the cooling water is 30 °C. The surface heat transfer coefficient is set as 1000, 2000, 3000, and 4000 W/(m^2^·°C) in the four control groups. The inner wall remains fixed at 600 W/(m^2^·°C).

For the linear elastic model, the incremental relationship between stress and strain is expressed in Equation (4).

(4) $$d\sigma =D_{e}d\varepsilon$$

where dσ is the stress increment, dε is the strain increment, and $D_{e}$ is the elastic modulus matrix.

As the material used in this simulation is stainless steel, its microstructure will not change. Therefore, the transformation strain increment in this simulation is 0. The strain increment can be expressed by the following Equation (5):

(5) $$d\varepsilon =d\varepsilon_{e}+d\varepsilon_{p}+d\varepsilon_{T}$$

where $d\varepsilon_{e}$, $d\varepsilon_{p}$ and $d\varepsilon_{T}$ are the elastic strain increment, the plastic strain increment, and the thermal strain increment.

When a particle in a stressed object is in a state of unidirectional stress, as long as the unidirectional stress reaches the yield point of the material, the particle begins to change from the elastic state to the plastic state. In other words, when the particle is in a state of multi-directional stress in a buckling object, all the stress components must be considered at the same time. Under certain deformation conditions, only when the relationship between the stress components is in accordance with a certain relationship does the particle begin to enter the plastic state. This is called the yield criterion, also known as the plastic condition. Mises’ yield criterion is adopted by ANSYS. The meaning is that when the element shape changes to a certain extent, the material begins to yield. It is represented by the following expression:

(6) $$\sqrt{\frac{1}{2}\left[{\left(\sigma_{r}-\sigma_{\theta }\right)}^{2}+{\left(\sigma_{r}-\sigma_{\theta }\right)}^{2}+{\left(\sigma_{z}-\sigma_{\theta }\right)}^{2}\right]\leq \sigma_{z}}$$

### 2.3. Verification of the Thermo-Mechanical Modeling Framework

**Figure 4:**
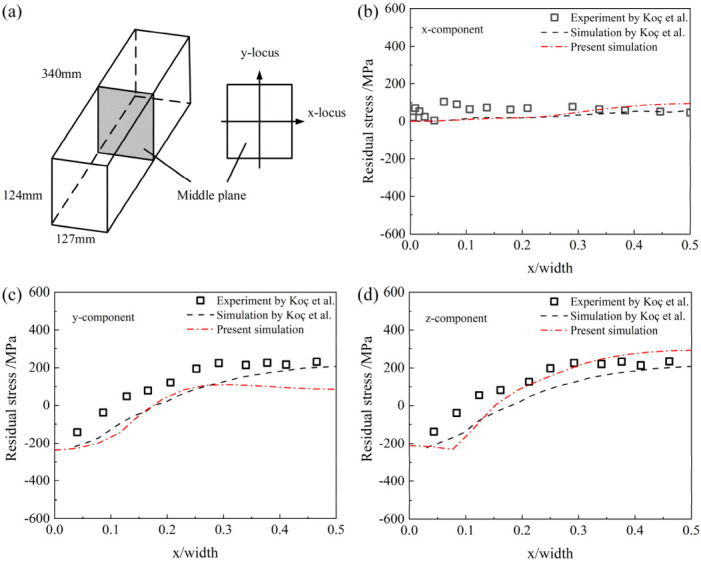
Calculated residual stresses compared to experimental [28] and the present study [24]. (**a**) model; (**b**) x-component; (**c**) y-component; (**d**) z-component.

In our previous research [24], the numerical methodology adopted in the present study was compared with experimental measurements, and satisfactory agreement was obtained. To further assess the reliability of the thermo-mechanical coupling procedure, an additional comparison was performed using the experimental data reported by Koç et al. [28], as shown in Figure 4. In the study of Koç et al. [28], the residual stresses generated during the quenching of an Al7050 aluminum alloy block were experimentally measured. The dimensions of the specimen were 340 mm × 127 mm × 124 mm. The residual stress distribution predicted by the present numerical framework is in reasonable agreement with the experimental results. It should be noted that Al7050 and AISI 310S possess significantly different thermophysical and mechanical properties. Therefore, the comparison presented here is intended to verify the numerical implementation and the thermo-mechanical coupling strategy rather than to provide a direct experimental validation of the material-specific response of AISI 310S. The agreement between the numerical and experimental results demonstrates the reliability of the adopted thermo-mechanical solution procedure.

## 3. Results and Discussion

### 3.1. Temperature Field

The temperature of the outer wall rapidly decreases during the quenching process. When the outer wall reaches the specified temperature, water-cooling stops, and the steel tube is subsequently cooled in air only.

**Figure 5:**
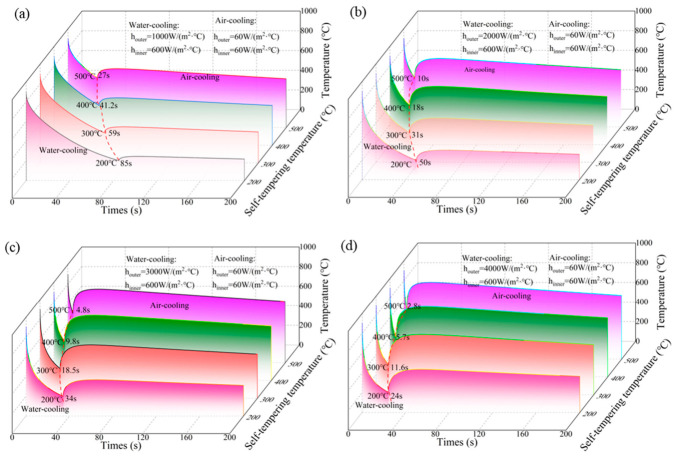
Temperature curves of the outer wall under different cooling intensities. (**a**) h_outer_ = 1000 W/(m^2^·°C); (**b**) h_outer_ = 2000 W/(m^2^·°C); (**c**) h_outer_ = 3000 W/(m^2^·°C); (**d**) h_outer_ = 4000 W/(m^2^·°C).

**Figure 6:**
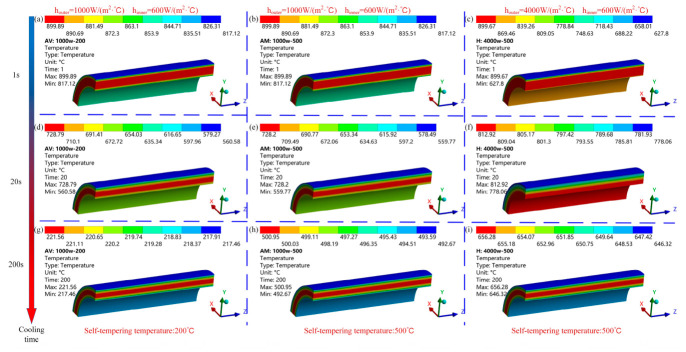
Temperature distribution of the seamless steel tube. (**a**) Self-tempering temperature: 200 °C, h_outer_ = 1000 W/(m^2^·°C), t = 1 s; (**b**) self-tempering temperature: 500 °C, h_outer_ = 1000 W/(m^2^·°C), t = 1 s; (**c**) self-tempering temperature: 500 °C, h_outer_ = 4000 W/(m^2^·°C), t = 1 s; (**d**) self-tempering temperature: 200 °C, h_outer_ = 1000 W/(m^2^·°C), t = 20 s; (**e**) self-tempering temperature: 500 °C, h_outer_ = 1000 W/(m^2^·°C), t = 20 s; (**f**) self-tempering temperature: 500 °C, h_outer_ = 4000 W/(m^2^·°C), t = 20 s; (**g**) self-tempering temperature: 200 °C, h_outer_ = 1000 W/(m^2^·°C), t = 200 s; (**h**) self-tempering temperature: 500 °C, h_outer_ = 1000 W/(m^2^·°C), t = 200 s; (**i**) self-tempering temperature: 500 °C, h_outer_ = 4000 W/(m^2^·°C), t = 200 s.

As can be seen from Figure 5, when water-cooling is followed by air-cooling, the surface temperature increases. This is called “self-tempering”. The initial temperature at which self-tempering occurs is called the self-tempering temperature. The outer wall of the steel tube is in direct contact with the cooling medium. In addition, there is a large temperature gradient between the steel tube and the cooling medium, which produces a large amount of heat transfer in a short time. In contrast, heat transfer within the steel tube occurs mainly through conduction. The heat transfer speed is slow, so the internal temperature is still at a high level. When the water-cooling is stopped, due to the huge temperature deviation between the center and the surface of the steel tube, the heat of the inner wall is gradually transferred to the outer wall, making the temperature of the outer wall rise whilst the temperature of the center drops. With the decrease in the temperature gradient of the steel tube in the direction of the wall thickness, the heating rate of the outer wall slows down. When it reaches the specified temperature, the heat transfer from the center to the outer wall is less than the heat transfer from the outer wall to the air convection, and the temperature of the outer wall gradually decreases. As shown in Figure 6a, the cooling time of the steel tube cooled from 500 °C to 400 °C was 14.2 s. However, when the temperature decreased from 400 °C to 300 °C, the cooling time increased to 17.8 s, which was 3.6 s longer than that for cooling from 500 °C to 400 °C. Further, the cooling time from 300 °C to 200 °C was 26 s, which increased by 8.2 s. The same phenomenon also appears in the temperature curves of the steel tube under different cooling intensities.

The increase in the cooling intensity of the outer wall decreases the time for the outer wall to cool to the specified temperature. However, the reduction rate gradually decreases. When the temperature reaches 500 °C, the cooling intensity of the outer wall (*h*): 1000→2000→3000→4000 W/(m^2^·°C), and the required time: 27→10→4.8→2.8 s. Furthermore, the reheating phenomenon becomes more obvious with the increase in cooling intensity. Take the case of the steel tube, which is water-cooled to 500 °C and air-cooled to the end, as an example. When the cooling intensity is 1000 W/(m^2^·°C), 2000 W/(m^2^·°C), 3000 W/(m^2^·°C) and 4000 W/(m^2^·°C), the temperature of the outer wall rises by 93 °C, 195 °C, 250 °C and 280 °C, respectively.

Figure 6 shows a cloud diagram of the temperature distribution in the thickness direction under different cooling intensities. In the initial stage, there is a large temperature gradient between the wall surface and the center under water-cooling conditions. Similarly, take the case where the steel pipe is water-cooled to 500 °C and air-cooled to the end as an example. When the cooling intensity is 1000 W/(m^2^·°C), the maximum temperature difference is about 82 °C. When the cooling intensity increases to 4000 W/(m^2^·°C), the maximum temperature difference increases to 280 °C. When t = 200 s, the temperature of each position of the steel tube is almost the same, and the maximum temperature deviation is within 10 °C.

### 3.2. Stress Field

**Figure 7:**
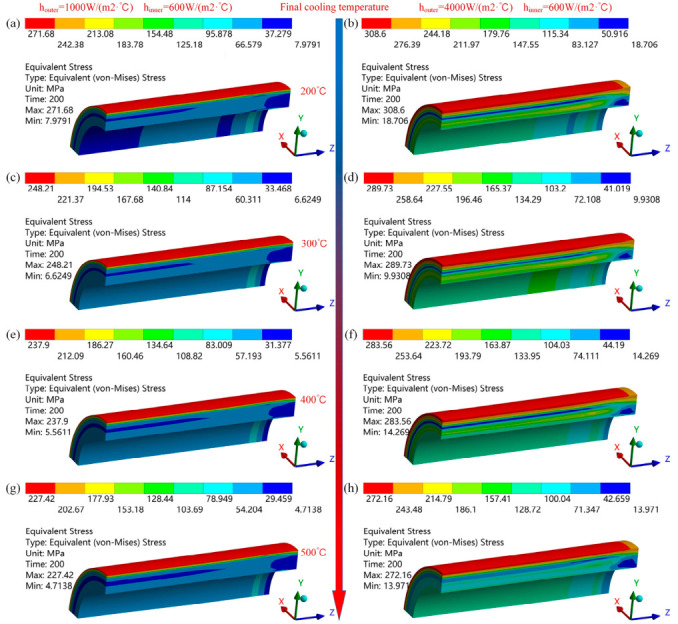
Equivalent stress distribution of the steel tube. (**a**) h_outer_ = 1000 W/(m^2^·°C), self-tempering temperature: 200 °C; (**b**) h_outer_ = 4000 W/(m^2^·°C), self-tempering temperature: 200 °C; (**c**) h_outer_ = 1000 W/(m^2^·°C), self-tempering temperature: 300 °C; (**d**) h_outer_ = 4000 W/(m^2^·°C), self-tempering temperature: 300 °C; (**e**) h_outer_ = 1000 W/(m^2^·°C), self-tempering temperature: 400 °C; (**f**) h_outer_ = 4000 W/(m^2^·°C), self-tempering temperature: 400 °C; (**g**) h_outer_ = 1000 W/(m^2^·°C), self-tempering temperature: 500 °C; (**h**) h_outer_ = 4000 W/(m^2^·°C), self-tempering temperature: 500 °C.

Figure 7 shows the equivalent stress distribution of the steel tube under different cooling intensities. The maximum equivalent stress is located at the outer wall, and the minimum value is near the center. The maximum equivalent stress decreases with the increase in self-tempering temperature.

Table 3 quantitatively summarizes the effects of the cooling intensity on the thermal and stress responses of the seamless steel tube. Increasing the outer-wall heat transfer coefficient from 1000 to 4000 W/(m^2^·°C) significantly accelerates the cooling process, reducing the time required for the outer wall to cool to 500 °C from 27 s to 2.8 s, corresponding to a reduction of approximately 89.6%. Meanwhile, the temperature recovery during the subsequent air-cooling stage increases from 93 °C to 280 °C, indicating that a larger cooling intensity produces a greater temperature gradient through the tube wall and therefore a stronger reheating effect. The maximum equivalent stress also increases with the cooling intensity, rising from 227 MPa at 1000 W/(m^2^·°C) to 272 MPa at 4000 W/(m^2^·°C). However, the increase gradually becomes less pronounced at higher cooling intensities. Specifically, the stress increases by 25 MPa when the cooling intensity increases from 1000 to 2000 W/(m^2^·°C), whereas it increases by only 5 MPa when the cooling intensity increases from 3000 to 4000 W/(m^2^·°C). This indicates that the sensitivity of the residual stress to the cooling intensity decreases at high cooling intensities. Therefore, although increasing cooling intensity improves cooling efficiency and promotes temperature recovery, it also increases thermal stress levels, suggesting that an appropriate compromise between cooling efficiency and stress control should be considered in industrial applications.

**Figure 8:**
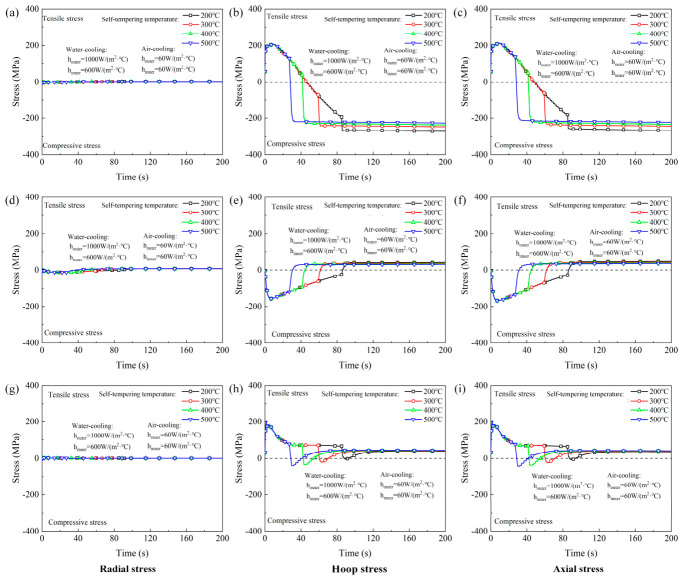
The change curve of each stress component. (**a**) Radial stress of the outer wall; (**b**) hoop stress of the outer wall; (**c**) axial stress of the outer wall; (**d**) radial stress of the center; (**e**) hoop stress of the center; (**f**) axial stress of the center; (**g**) radial stress of the inner wall; (**h**) hoop stress of the inner wall; (**i**) axial stress of the inner wall.

In Figure 8, “+” represents tensile stress, and “–” represents compressive stress. The radial stress is much smaller than the axial and hoop stress components. In the early stage, the steel tube is in the water-cooling stage. The outer wall is in a tensile stress state. The center is in a compressive stress state, and its value gradually decreases after a rapid rise. After water-cooling, the steel tube enters the air-cooling stage, and the stress states change. The outer wall of the steel tube changes from tensile stress to compressive stress. The stress of the center changes from compressive stress to tensile stress. The stress of the inner wall changes from tensile stress to compressive stress. With the increase in the air-cooling time, the stress component of the outer wall and the center does not obviously change. However, the stress component of the inner wall gradually decreases with the increase in the air-cooling time, and then the stress changes from compressive stress to tensile stress. In addition, with the increase in the self-tempering temperature, the stress components of the outer wall decrease, but the stress components of the center are almost the same as those of the inner wall.

**Figure 9:**
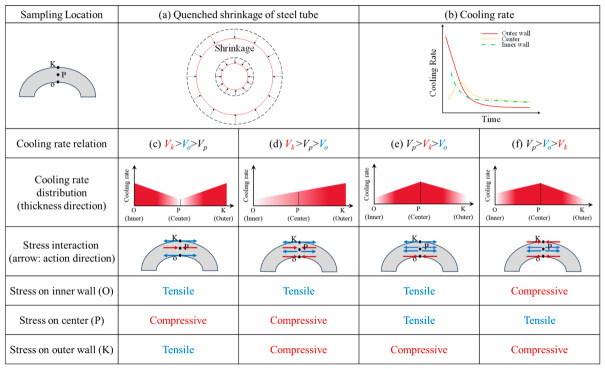
Schematic diagram of the relationship between cooling rate and stress during cooling. (**a**) Quenched shrinkage of steel tube; (**b**) cooling rate; (**c**) *V_k_* > *V_o_* > *V_p_*; (**d**) *V_k_* > *V_p_* > *V_o_*; (**e**) *V_p_* > *V_k_* > *V_o_*; (**f**) *V_p_* > *V_o_* > *V_k_*.

Figure 9 shows the change in the interaction direction of stress at each position with the cooling rate. O, P, and K represent the inner wall, center, and outer wall of the steel tube, respectively. During cooling, the steel tube shrinks as a whole. With the increase in cooling time, the cooling rate of each position changes. When *V_k_* >*V_o_* >*V_p_*, the shrinkage of the outer wall and inner wall is much greater than that of the center. Therefore, the outer wall and the inner wall face the center of the steel tube to apply compressive stress. According to the interaction of the stress, the center of the steel tube hinders the shrinkage of the outer wall and the inner wall and applies tensile stress to both. When *V_k_* >*V_p_* >*V_o_*, the outer wall faces the center with a compressive stress, while the inner wall faces the center with a tensile stress. When *V_p_* > *V_k_* > *V_o_*, both the outer and inner walls exert tensile stress on the center of the steel tube. When *V_p_* > *V_o_* > *V_k_*, the relationship between the cooling rate between the inner and outer walls and the center has not changed. Therefore, the stress direction does not change.

**Figure 10:**
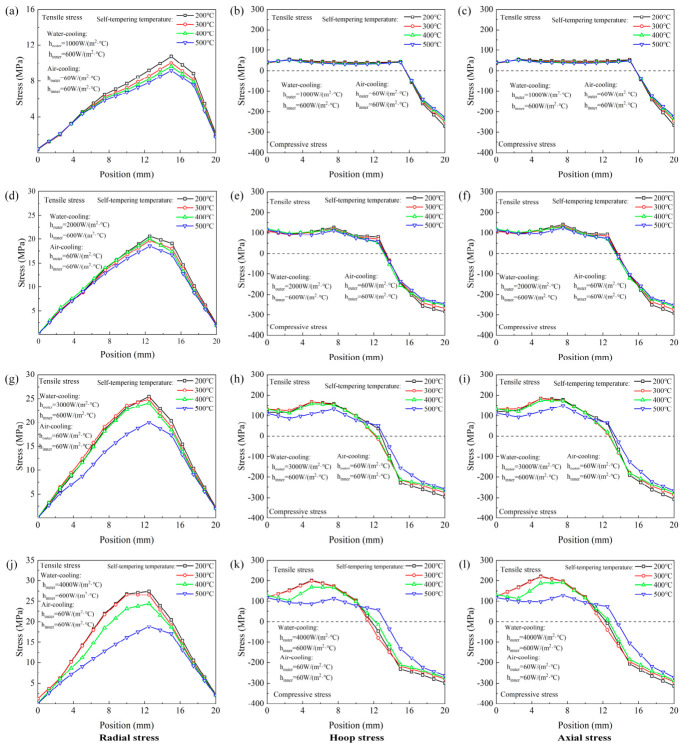
Stress distribution in the thickness direction of the steel tube. (**a**) Radial stress of the outer wall at h_outer_ = 1000 W/(m^2^·°C); (**b**) hoop stress of the outer wall at h_outer_ = 1000 W/(m^2^·°C); (**c**) axial stress of the outer wall at h_outer_ = 1000 W/(m^2^·°C); (**d**) radial stress of the center at h_outer_ = 2000 W/(m^2^·°C); (**e**) hoop stress of the center at h_outer_ = 2000 W/(m^2^·°C); (**f**) axial stress of the center at h_outer_ = 2000 W/(m^2^·°C); (**g**) radial stress of the inner wall at h_outer_ = 3000 W/(m^2^·°C); (**h**) hoop stress of the inner wall at h_outer_ = 3000 W/(m^2^·°C); (**i**) axial stress of the inner wall at h_outer_ = 3000 W/(m^2^·°C); (**j**) radial stress of the inner wall at h_outer_ = 4000 W/(m^2^·°C); (**k**) hoop stress of the inner wall at h_outer_ = 4000 W/(m^2^·°C); (**l**) axial stress of the inner wall at h_outer_ = 4000 W/(m^2^·°C).

**Figure 11:**
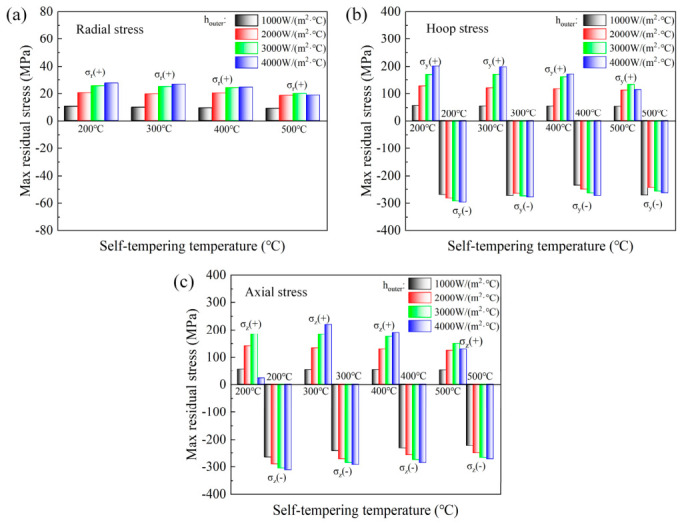
Maximum values of stress components at different cooling intensities. (**a**) Radial stress; (**b**) hoop stress; (**c**) axial stress.

The radial stress of the steel tube is tensile stress, and the stress value gradually decreases with the increase in the self-tempering temperature (as shown in Figure 10). With the increase in the self-tempering temperature, the position of the stress peak gradually shifts to the outer wall. The same variation rule can be seen in the axial stress distribution and the hoop stress distribution. When the cooling intensity of the outer wall is 1000 W/(m^2^·°C), there is a large compressive stress. The distribution range of the compressive stress is small and mainly concentrated near the outer wall. Although the value of the tensile stress is smaller than the compressive stress, the distribution range is wider. With the increase in the cooling intensity of the outer wall, the distribution range of the compressive stress increases. Correspondingly, the distribution range of the tensile stress gradually decreases, but its magnitude significantly increases. The maximum compressive stress occurs at the outer wall, while the maximum tensile stress occurs near the inner wall. With the increase in the cooling intensity, the maximum stress gradually increases, as shown in Figure 11. However, with the increase in the self-tempering temperature, the maximum values of compressive stress and tensile stress gradually decrease.

### 3.3. Limitations of the Present Model

(1) In the present model, phase transformation effects were neglected. Therefore, the predicted stress field only represents thermal stress contributions.
(2) The heat transfer coefficient of the inner wall was assumed constant (600 W/m^2^·K), whereas industrial cooling conditions may vary with water flow rate and spray characteristics.
(3) Future studies will incorporate phase transformation kinetics and variable cooling boundary conditions.

## 4. Conclusions

In this study, the temperature-controlled quenching process of a hot-rolled seamless steel tube was simulated by ANSYS. Since the seamless steel tube is an axisymmetric model, only a 1/8 model was used in the physical model to simplify the calculation. In the process of simulation, the cooling intensity and the self-tempering temperature are the variables. The cooling intensity of the outer wall was set to four values: 1000, 2000, 3000, and 4000 W/(m^2^·°C). The cooling intensity of the inner wall remains unchanged at 600 W/(m^2^·°C). The self-tempering temperature was also set to four values: 200, 300, 400, and 500 °C. The temperature change curves under different cooling intensities were obtained. The temperature return phenomenon of the steel tube under different self-tempering temperatures was analyzed. According to the stress variation curves of the outer wall, the center, and the inner wall of the steel tube, the distribution law of each stress component with the cooling intensity was obtained.

At the same self-tempering temperature, the greater the cooling intensity of the outer wall surface of the steel tube, the faster the temperature drop and the greater the return temperature. The maximum value of equivalent stress appears at the outer wall, while the equivalent stress near the center is the smallest. The maximum equivalent stress decreases with the increase in the self-tempering temperature.

The radial stress of the steel tube is tensile stress, and the stress value gradually decreases with the increase in the self-tempering temperature. With the increase in the self-tempering temperature, the position of the stress peak gradually shifts to the outer wall. With the increase in the cooling intensity of the outer wall, the distribution range of the compressive stress increases. Correspondingly, the distribution range of the tensile stress gradually decreases, but its magnitude significantly increases.

In the direction of thickness, the axial and hoop stress components show the distribution state of “internal tension and external compression”. With the increase in cooling intensity, the maximum stress gradually increases. However, the increasing trend gradually decreases. In addition, with the increase in self-tempering temperature, the maximum values of compressive stress and tensile stress gradually decrease.

For industrial implementation, increasing the cooling intensity can improve the cooling efficiency but simultaneously increases the residual stress. Therefore, a compromise between cooling efficiency and residual stress control should be considered. Self-tempering temperatures above 400 °C are recommended to reduce residual stress levels.

## Figures and Tables

**Table 1:** Chemical composition % of 310S steel (mass fraction).

C	Si	Mn	Ni	Cr	S	P
≤0.08	≤1.5	≤2.0	19.0~22.0	24.0~26.0	≤0.03	≤0.045

**Table 2:** Thermophysical parameters of 310S steel.

Temperature/°C	Density /kg/m^3^	Coefficient of Thermal Expansion/1 × 10^−5^/°C	Young’s Modulus/GPa	Poisson’s Ratio	Thermal Conductivity /W/(m·K)
30	7931	1.65	208.5	0.3	12.8
100	7903	1.67	203.9	0.3	13.7
200	7863	1.69	197.1	0.3	14.9
300	7822	1.72	189.9	0.3	16.2
400	7780	1.74	182.5	0.3	17.5
500	7738	1.77	174.6	0.3	18.8
600	7695	1.79	166.5	0.3	19.9
700	7650	1.83	155.8	0.3	21.3
800	7600	1.89	144.8	0.3	22.9
900	7546	1.95	134.6	0.3	24.6

**Table 3:** Quantitative comparison of thermal and stress responses under different cooling intensities.

Outer Wall HTC/W/(m^2^·°C)	Cooling Time to 500 °C/s	Temperature Recovery/°C	Maximum Equivalent Stress/MPa	Stress Increase Rate/%
1000	27	93	227	0
2000	10	195	252	11
3000	4.8	250	267	17.6
4000	2.8	280	272	19.8

## Data Availability

The original contributions presented in this study are included in the article. Further inquiries can be directed to the corresponding authors.
